# Immunoinflammatory evidence of rheumatoid arthritis caused by COVID-19

**DOI:** 10.1186/s40659-025-00620-7

**Published:** 2025-06-10

**Authors:** Zhiqiang Shao, Dan Xia, Liang Zhou, Zonghan Xu, Jiaqian Wang

**Affiliations:** 1 Department of Orthopedic, Suzhou Municipal Hospital, The Affiliated Suzhou Hospital of Nanjing Medical University, Gusu School of Nanjing Medical University, Suzhou, 215008 China; 2 Department of Respiratory, Affiliated Wuxi Fifth Hospital of Jiangnan University, Wuxi, 214000 China; 3 Department of Orthopedic, Huai’an Hospital Affiliated to Yangzhou University, The Fifth People’s Hospital of Huai’an, Huai’an, 223399 China; 4 Department of Orthopedic, Zhongshan Hospital, Fudan University, Shanghai, 200032 China

**Keywords:** COVID-19, Rheumatoid arthritis, Immune cells, Mendelian randomization, Meta-analysis

## Abstract

**Purpose:**

The relationship between coronavirus disease 2019 (COVID-19) and rheumatoid arthritis (RA) remains uncertain. We aimed to assess the association between COVID-19 and RA through immune inflammation.

**Methods:**

First, we conducted a meta-analysis on the risk of COVID-19 infection, hospitalization rate, and mortality rate for patients with RA. Then, Mendelian randomization (MR) was used to evaluate the causal relationship between COVID-19 and RA, and further analyzed the cytokines and immune cells in COVID-19 and RA. Finally, we obtained microarray datasets of COVID-19, RA patients, and normal controls from the GEO database. And performed functional, pathway enrichment, and immune cell infiltration analysis on differentially expressed genes between each group.

**Results:**

The meta-analysis results suggested that the hospitalization rate and mortality rate of RA patients infected with COVID-19 were higher than those of the control population. MR analysis showed a positive correlation between COVID-19 infection and RA. We also found that interleukin 13 was associated with RA and COVID-19 infection. CD27 on IgD + CD24 + B cells and CD3 on CD39 + CD8 + T cells are common immune cell phenotypes in two diseases. In addition, COVID-19 function is enriched in immune responses mediated by leukocytes and neutrophils, while RA is significantly enriched in the proliferation of T and B lymphocytes. The results of immune cell infiltration showed that both diseases had more neutrophils and fewer CD8 T cells.

**Conclusion:**

There are many similarities between COVID-19 and RA in immune inflammatory responses such as cytokines and immune cells. COVID-19 may lead to the development of RA through immune inflammation.

**Supplementary Information:**

The online version contains supplementary material available at 10.1186/s40659-025-00620-7.

## Introduction

The coronavirus disease 2019 (COVID-19) is an infectious disease caused by severe acute respiratory syndrome coronavirus 2 (SARS-CoV-2), which is a global pandemic, bringing heavy burden to global health and society [1]. COVID-19 mainly presents with respiratory symptoms such as fever, dry cough and fatigue, but also involves multiple organs such as digestive tract, nervous system, immune system, musculoskeletal system, etc [2]. According to the previous statistics, 27.3% of the cases had musculoskeletal complaints of Myalgia and Arthralgia after infection with COVID-19 [3]. Generally speaking, viruses can trigger arthritis through direct colonization at the joints or through abnormal immune inflammatory reactions produced by the host during the infection response [4]. Viral arthritis in COVID-19 has been described in many case reports, but its significance as a new clinical entity has not been fully recognized.

Inflammatory arthritis is a chronic inflammatory disease characterized by joint pain in the joints, muscles, and surrounding soft tissues, mainly including rheumatoid arthritis (RA), spondyloarthritis (SpA) [5]. The etiology of RA is not fully understood, but it is highly correlated with genetic and immune factors. Genetically, RA has a genetic predisposition, with a significantly higher frequency of abnormalities in the HLA-DRB1 gene compared to the normal population [6]. More than 100 genes, including STAT4, PADI4, PTPN22, etc., are closely related to the pathogenesis of RA [7]. From immune perspective, several pro-inflammatory cytokines such as tumor necrosis factor α and interleukin (IL) play a promoting role in the development of RA [8]. The increase of IL-1β and IL-6 can induce CD4 + T cells to differentiate into activated Th1 and Th17 cells [9]. The immune activation pattern of COVID-19 patients, such as the secretion of proinflammatory mediators and T cell activation, seems to be similar to the immune response of RA [10]. Derksen found that 3 of 61 COVID-19 patients developed polyarthritis similar to RA after infection [11]. RA patients are also more likely than the general population to have serious COVID-19 infection, and the infection rate is about twice that of the general population [12]. Although many cases of inflammatory arthritis after SARS-CoV-2 infection have been reported in the existing clinical practice, more information is needed to know whether there is a correlation or just a coincidence between the two.

Emerging evidence suggests that SARS-CoV-2 infection can attack the musculoskeletal system through immune inflammatory mechanisms, which may develop into inflammatory arthritis in the infection or post infection stage [13]. MR has been widely used for causal inference in epidemiology [14]. The main principle of this method is to study the impact of exposure factors on the results by using genotypes instead of exposure factors, which can reduce confounding bias [15]. Because little is known about the manifestations of arthritis caused by COVID-19 infection, and the musculoskeletal manifestations are phenotypically similar to inflammatory arthritis, we tried to find the relationship between COVID-19 and RA through MR and bioinformatics analysis of immune inflammatory components. By integrating various analysis methods, we can determine whether COVID-19 will lead to the progression of RA, and whether RA patients are more likely to be infected with COVID-19.

## Materials and methods

### Meta-analysis

#### Search strategy and eligibility criteria

This work was conducted in accordance with the Preferred Reporting Items for Systematic Reviews and Meta-Analyses (PRISMA) Statement. We searched PubMed, Embase, and Cochrane Library, all set for the search period from December 1, 2019 to August 31, 2023. The following search terms were used individually or in combination: “COVID-19” or " SARS-CoV-2” and " rheumatoid arthritis”. We did not impose any language restrictions on our search.

We reviewed all the retrieved abstracts and full texts. The research criteria included in this analysis were as follows: (1) COVID-19 infection in patients with RA and their control population; (2) Prevalence of RA in COVID-19 patients and control population; (3) The patient’s various types of information are detailed and complete; (4) Analytical studies include cohort studies and case-control studies, excluding reviews, case reports, and animal experiments.

### Outcome measures

The main purpose of this study is to observe whether there is a statistical difference in the number of COVID-19 infections between RA patients and their control population. And whether there is a difference in the prevalence of RA between patients with COVID-19 and non-COVID-19 control population. The severity of infection is determined by the number of inpatients and deaths after COVID-19 infection. The number of severe infections and the number of ICU hospitalizations are secondary criteria. We also analyzed the antibody production of RA patients and control population after COVID-19 vaccination.

### Statistical analysis

This work was performed using Revman 5.3 software (Nordic Cochran Centre, Copenhagen, Denmark). We used the mantel–Haenszel method for binary data. Select the risk ratio (RR) as the comprehensive effect quantity indicator, and provide point estimates and 95% CI for each effect quantity. The I2 statistic was used to assess heterogeneity in the assay (value 50% or higher for high heterogeneity). All *p* values < 0.05 were considered statistically significant.

### Mendelian randomization

#### Data sources

The genetic variation data related to COVID-19 came from GWAS, and the gene instruments variable is composed of related single nucleotide polymorphism (SNPs) sites. COVID-19 and RA were subjected to MR analysis as exposure and outcome, respectively. The SNPs of COVID-19 included SARS CoV-2 infection (38984 cases and 1644784 controls), COVID-19 hospitalization (9986 cases and 1877672 controls) and severe COVID-19 (5101 cases and 1383241 controls). SARS-CoV-2 infection was defined as a laboratory confirmed SARS-CoV-2 infection, with or without symptoms. COVID-19 hospitalization refers to the first hospitalization between 7 days before the positive date of COVID-19 and 15 days after the positive date of COVID-19 for the first time. Severe COVID-19 is defined as dyspnea, respiratory rate ≤ 30/min, SpO2 ≤ 93%, PaO2/FiO2 < 300mmHg or lung field infiltration exceeding 50%. The detailed information on summarizing GWAS results of COVID-19 is shown in Supplemental Table 1.

The data on RA is sourced from the IEU database. Detailed information on RA, such as the number of cases and controls, is also listed in Supplemental Table 1. The SNPs of inflammatory cytokines and immune cell characteristics was derived from previously published MR analysis [16, 17]. Inflammatory cytokines include a total of 91 plasma proteins, such as chemokines, interleukin, tumor necrosis factor, etc. (GCST90274758 to GCST90274848). Immune cells include 731 phenotypes, including B cells, T cells, natural killer cells, monocytes, etc. (GCST90001391 to GCST90002121).

### Defining genetic instruments

The genetic instruments selected by MR needs to meet three important prerequisites, namely, the genetic tool is highly correlated with exposure factors (relevance), but not with confounding factors (independence), and can only affect the results through exposure (exclusion-restriction) [18]. Based on the above three assumptions, obtain genetic variations strongly (*p* < 5 × 10^–8^) and independently (r^2^ < 0.01) associated with COVID-19 and RA. The physical distance between genes kb = 10,000. The included SNPs were statistically analyzed by various methods to infer the causal relationship between COVID-19 and RA. And further explore the immune inflammatory connection between COVID-19 and RA through SNPs of inflammatory cytokines and immune cells.

### Statistical analysis

The main statistical method used to infer the causal effect between COVID-19 and RA is the inverse variance weighted (IVW) method. MR analysis was based on genetic variants as IVs to estimate the causal association of exposure on outcome. MR – Egger method, weighted median method, simple mode method, and weighted mode method are used to supplement MR results. Evaluate the sensitivity of results using MR Egger intercept, Cochran Q test, and leave-one-out method. By removing SNPs one by one and calculating the effects of merging other units, the effect of a single SNP on outcomes is clarified, and the degree of impact and stability are evaluated [19]. The causal relationship between immune inflammatory components and the two diseases was analyzed using the same method. All analyses were carried out using the “TwoSampleMR” and “MRPRESSO” packages in R version 4.0.3, with a statistically significant *p* < 0.05. And false discovery rate (FDR) control was performed on the p-values of inflammatory cytokines and immune cells, with qval < 0.05 having statistical significance.

### Bioinformatics analysis

#### Datasets download

We used “rheumatoid arthritis” and “COVID-19” as keywords to search related datasets in GEO database (https://www.ncbi.nlm.nih.gov/geo/). The inclusion criteria are as follows: (1) Homo sapiens microarray analysis of RA and COVID-19 with complete data; (2) Tissue samples were taken from the patient’s whole blood; (3) Number of tissue samples should be at least 80. We ultimately obtained two datasets that met the criteria. GSE213313 was compared with 83 COVID-19 patients and 11 normal controls. Among them, 36 patients were admitted to the intensive care unit and received invasive mechanical ventilation, 15 patients received supplemental oxygen in the regular medical ward, and 8 patients died. No patients received corticosteroids or other potent anti-inflammatory treatments. GSE17755 included 112 RA patients and 45 normal controls for comparison. RNA samples from healthy volunteers matched with patients by age and gender were used as controls.

### Identification and functional enrichment analysis of DEGs

Differential analysis was conducted using the “Limma” software package, which is a screening method for differentially expressed genes (DEGs) based on a generalized linear model. The FDR value less than 0.05 and |log2-fold change (FC)| > 1.2 were considered to be statistically significant. The results of DEGs are presented by volcano map and heatmap. Cluster analysis of intra group data repeatability using principal component analysis (PCA) on the RA and COVID-19 datasets. Specifically, we used the R software package “Stats” to score the expression profile and further use the “prcomp” function for dimensionality reduction analysis to obtain the matrix.

We conducted enrichment analysis on DEGs between COVID and control, DEGs between RA and control, and common DEGs between the two to improve accuracy. We obtained the latest Gene Ontology (GO) and Kyoto Encyclopedia of Genes and Genomes (KEGG) pathway gene annotations as background, mapped genes to the background set, and performed enrichment analysis using the R software package “clusterProfiler” to obtain the results of enrichment analysis. *P* < 0.05 is considered to be statistically significant. The enrichment pathways and functions were visualized by “ggplot2” package.

### Evaluation of immune cells

Based on the expression profile file, we used the R software package “IOBR” to select the CIBERSORT and EPIC methods to calculate the immune infiltration cell score for each sample. CIBERSORT can transform the standardized gene expression matrix into the composition of invasive immune cells. This method uses LM22 feature matrix to define 22 components of infiltrating immune cells. EPIC is also based on deconvolution algorithm, analyzing the infiltration ratio of 8 types of immune cells based on expression data, and it can also compare different immune cells. The violin diagrams are used for visualization, and paired t-tests are used to evaluate significant differences between two groups.

## Results

### Selection of studies for meta-analysis

In total, we identified 883 articles through searching the databases, and then deleting duplicate articles. After scanning the title and abstract, 452 unrelated articles were excluded. Subsequently, through the review of full texts, 34 articles remained (**Supplementary references**). The basic process and results of literature screening are shown in Supplemental Fig. 1. Of the 34 studies, 3 compared the COVID-19 infected population with the control population, and the remaining 31 compared RA with the control population. The country with the most data sources included in the study is the United States, and the basic characteristics of the included studies are detailed in Supplemental Tables 2,**3**.

### Meta-analysis of RA in COVID-19 patients

To further evaluate the relationship between COVID-19 and RA, we compared the differences in the prevalence of RA between COVID-19 infection and control populations. This comparison included three RA studies, with data analyzed as binary variables. The results showed that there was no statistically significant difference in the prevalence of inflammatory arthritis between COVID-19 patients and the control population (*p* = 0.41, I^2^ = 0%) Supplemental Fig. 2).

### Meta-analysis of COVID-19 in patients with RA

**Fig. 1:**
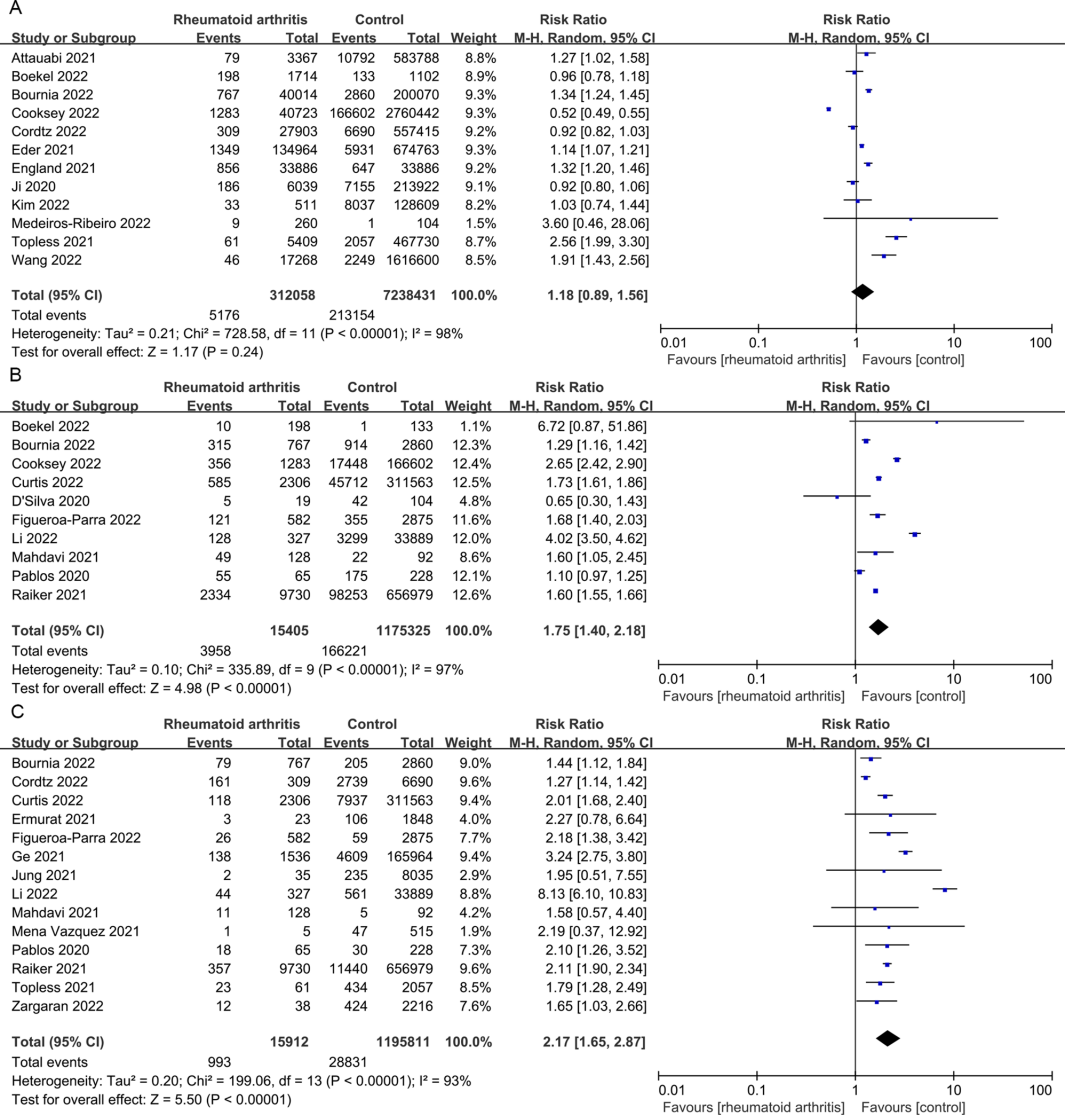
Prevalence, hospitalization and mortality of COVID-19 in patients with RA. (**A**) Forest plot of SARS-CoV-2 infection risk in patients with RA; (**B**) Forest plot of COVID-19 hospitalization rate in patients with RA; (**C**) Forest plot of COVID-19 mortality rate in patients with RA

**Fig. 2:**
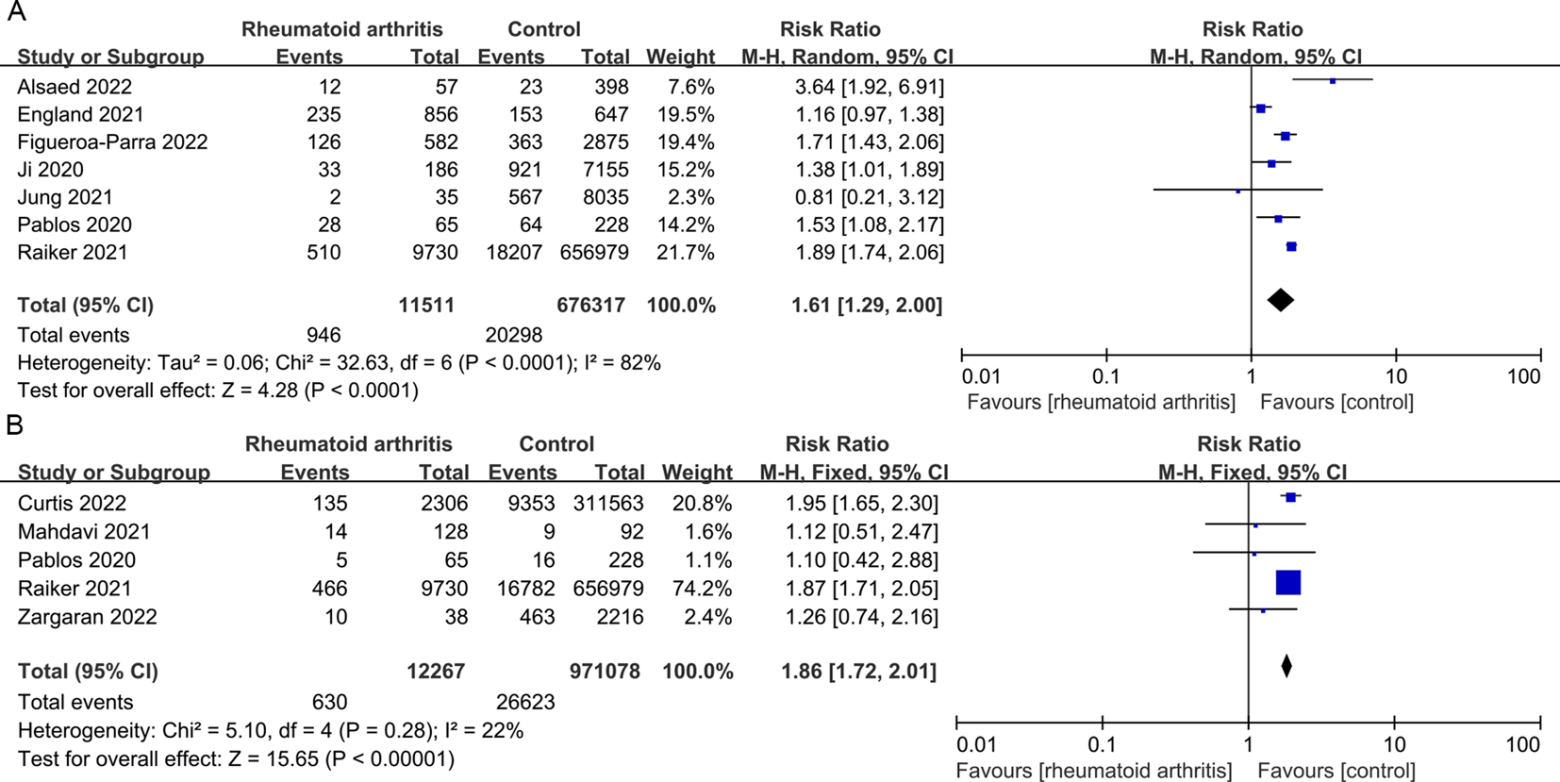
Severe infections and ICU hospitalization of COVID-19 in patients with RA. (**A**) Forest plot of COVID-19 severe infections in patients with RA; (**B**) Forest plot of COVID-19 ICU hospitalizations in patients with RA

At the same time, we conducted a bidirectional meta-analysis to compare the risk of COVID-19 infection between patients with RA and the control population. Firstly, we found no significant difference in the infection rate of COVID-19 between the experimental group and the control group (*p* = 0.24, I^2^ = 98%) (Fig. 1A). Secondly, we evaluated whether patients with RA are more likely to lead to adverse outcomes. We found that the hospitalization rate and mortality rate of COVID-19 infection in patients with RA were significantly higher than those in control patients (*p* < 0.00001 and *p* < 0.00001) (Fig. 1B-C). The number of severe infections and the number of ICU hospitalizations also show the same trend (*p* < 0.00001) (Fig. 2A-B).

Finally, we analyzed 9 items of data including the antibody positive rate of RA patients and non-RA control population after COVID-19 vaccination. The results showed that the antibody positive rate of RA patients after two doses of COVID-19 vaccine was significantly lower than that of the control population (*p* < 0.00001, I^2^ = 34%), and there was only slight heterogeneity among the studies (Supplemental Fig. 3).

### Causal effect of COVID-19 on RA

**Fig. 3:**
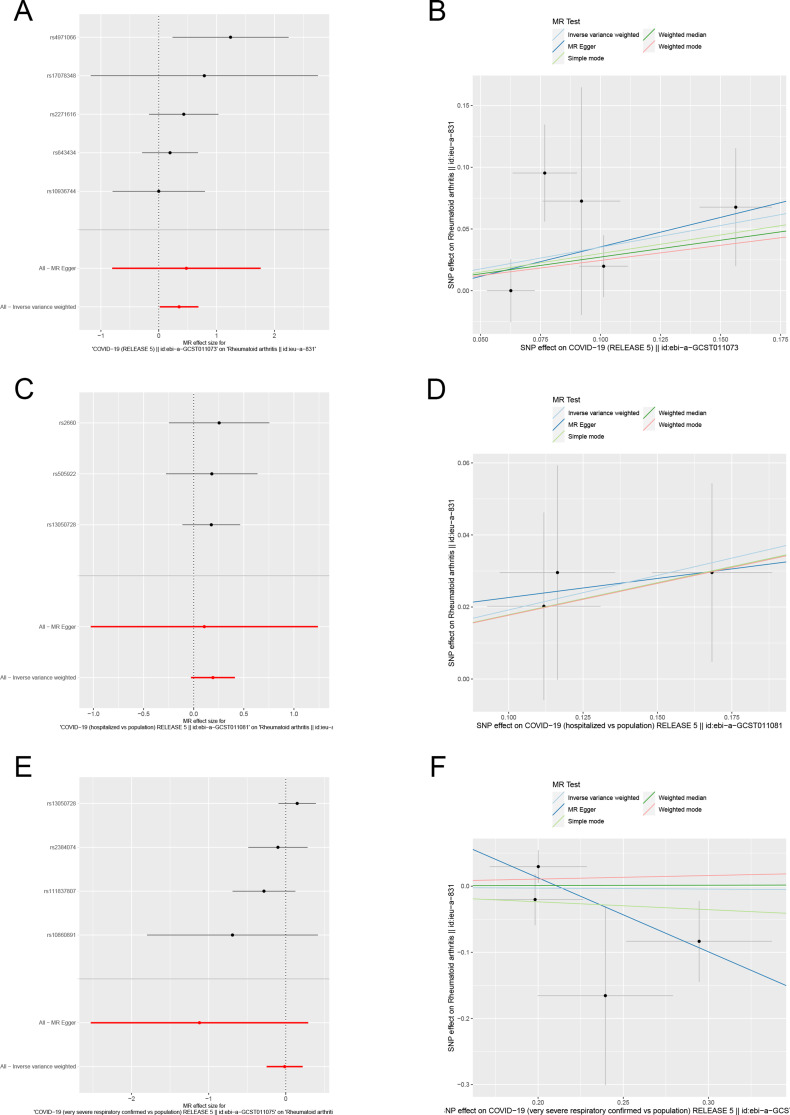
Forest plot and scatter plot for the causal relationship between COVID-19 and RA using different MR methods. (**A**) Forest plot of the causal relationships between SARS-CoV-2 infection and RA; (**B**) Scatter plot of the causal relationships between SARS-CoV-2 infection and RA; (**C**) Forest plot of the causal relationships between COVID-19 hospitalization and RA; (**D**) Scatter plot of the causal relationships between COVID-19 hospitalization and RA; (**E**) Forest plot of the causal relationships between severe COVID-19 and RA; (**F**) Scatter plot of the causal relationships between severe COVID-19 and RA. The slope of each line corresponds to the causal estimates for each method

We identified robust SNPs as instrumental variables for RA, respectively, after excluding pleiotropic SNPs. In the MR analysis using the IVW method, RA has a positive causal relationship with SARS-CoV-2 infection (beta = 0.3521, se = 0.1708, *p* = 0.03925), but is not associated with COVID-19 hospitalization or Severe COVID-19 (Fig. 3). In addition, the results of the leave-one-out method showed that after gradually removing each SNP, the results of the remaining SNPs were similar to the original results. The *p*-values of MR Egger intercept and Cochran Q heterogeneity are also > 0.05, indicating that the MR analysis results are reliable (Supplemental Fig. 4). In summary, these results indicate that from a genetic perspective, COVID-19 may increase the risk of RA to some extent.

### Causal effect of RA on COVID-19

Similarly, we identified robust SNPs as instrumental variables for COVID-19 infection, COVID-19 hospitalization and Severe COVID-19, respectively. The IVW results showed no causal relationship between RA and COVID-19 (Supplemental Fig. 5). The sensitivity analysis of the leave-one-out method and funnel plot did not find SNPs that had a strong influence on the results (Supplemental Fig. 6). These results suggest that RA does not cause COVID-19 genetically.

### Effects of cytokines on COVID-19 and RA

**Fig. 4:**
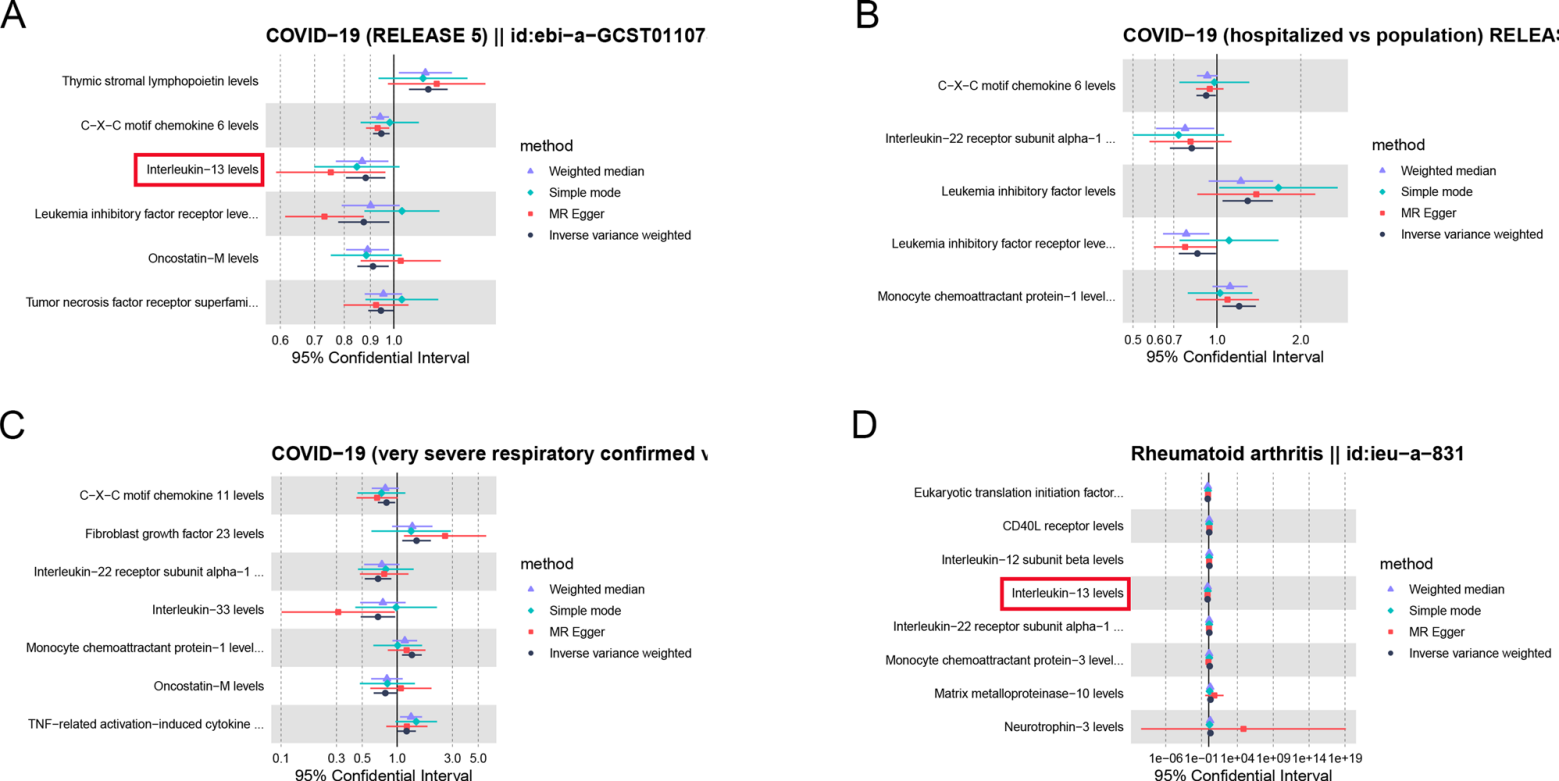
Effect of cytokines on COVID-19 and RA. (**A**) Forest plot of the causal relationships between SARS-CoV-2 infection and significant cytokines; (**B**) Forest plot of the causal relationships between COVID-19 hospitalization and significant cytokines; (**C**) Forest plot of the causal relationships between severe COVID-19 and significant cytokines; (**D**) Forest plot of the causal relationships between RA and significant cytokines

In order to further study the relationship between COVID-19 and RA in immune inflammation, we identified cytokines associated with COVID-19 and RA using the IVW method. Six cytokines have causal relationships with SARS CoV-2 infection, five cytokines have relationships with COVID-19 hospitalization, and seven cytokines have relationships with severe COVID-19 (Fig. 4A-C). C-X-C motif chemokine 6 is the same cytokine in SARS CoV-2 infection and COVID-19 hospitalization. Among RA related cytokines, IL-13 is the same cytokine in SARS CoV-2 infection (Fig. 4D).

### Effects of immune cells on COVID-19 and RA

**Fig. 5:**
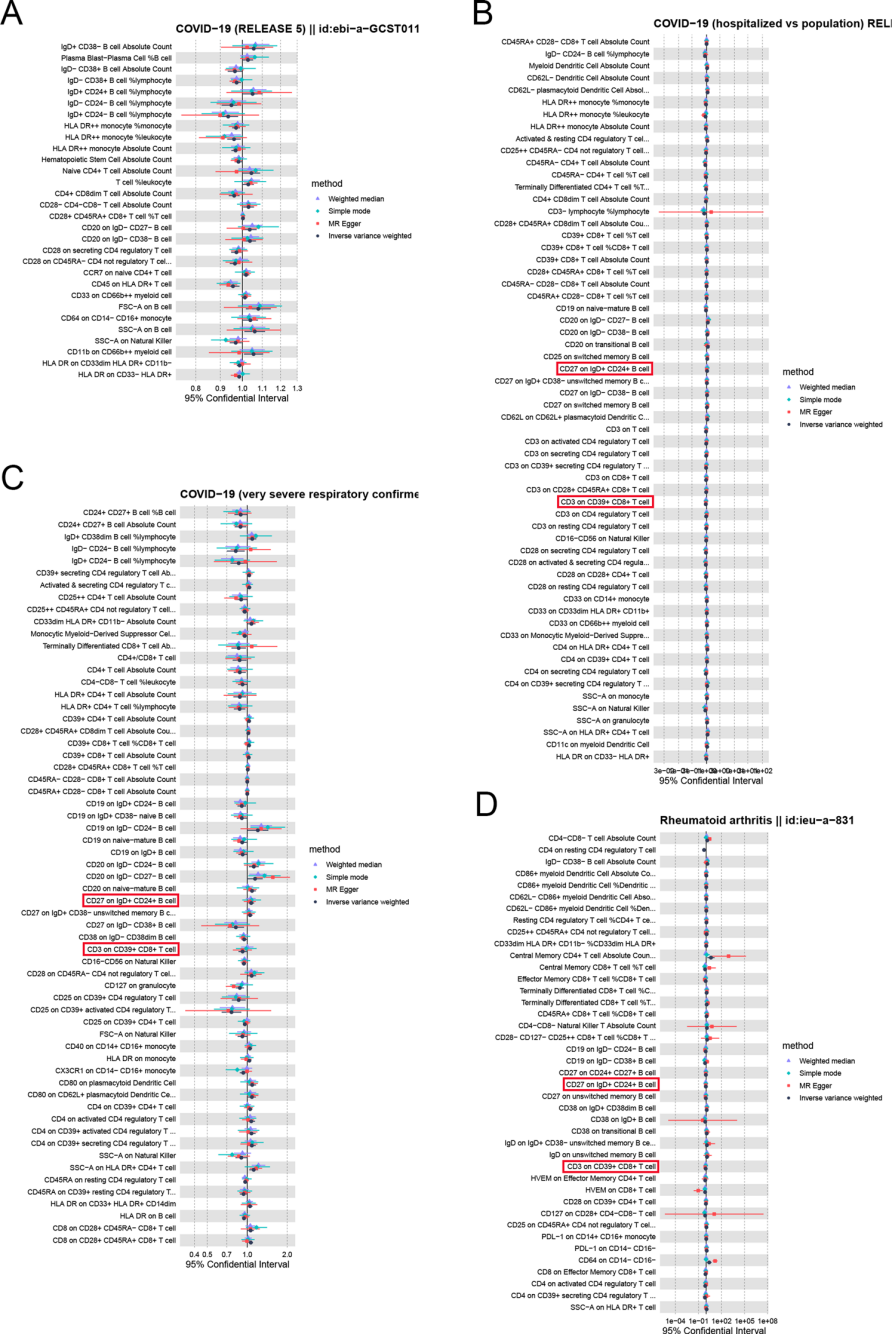
Effect of immune cells on COVID-19 and RA. (**A**) Forest plot of the causal relationships between SARS-CoV-2 infection and significant immune cells phenotype; (**B)** Forest plot of the causal relationships between COVID-19 hospitalization and significant immune cells phenotype; (**C**) Forest plot of the causal relationships between severe COVID-19 and significant immune cells phenotype; (**D**) Forest plot of the causal relationships between RA and significant immune cells phenotype

We carried out the same MR analysis of immune cells and identified immune cells related to COVID-19 and inflammatory arthritis. A total of 30 immune cells phenotypes were identified in SARS CoV-2 infection, 60 immune cells were identified in COVID-19 hospitalization, and 62 immune cells were identified in severe COVID-19 (Fig. 5A-C). CD28 + CD45RA + CD8 + T cell %T cell, IgD- CD24- B cell %lymphocyte and CD20 on IgD- CD27- B cell are the common immune cells phenotype among them. RA and COVID-19 hospitalization, severe COVID-19 have two same immune cells phenotypes (CD27 on IgD + CD24 + B cell and CD3 on CD39 + CD8 + T cell), but it does not have the same immune cells as SARS CoV-2 infection (Fig. 5D).

#### Identification of DEGs

**Fig. 6:**
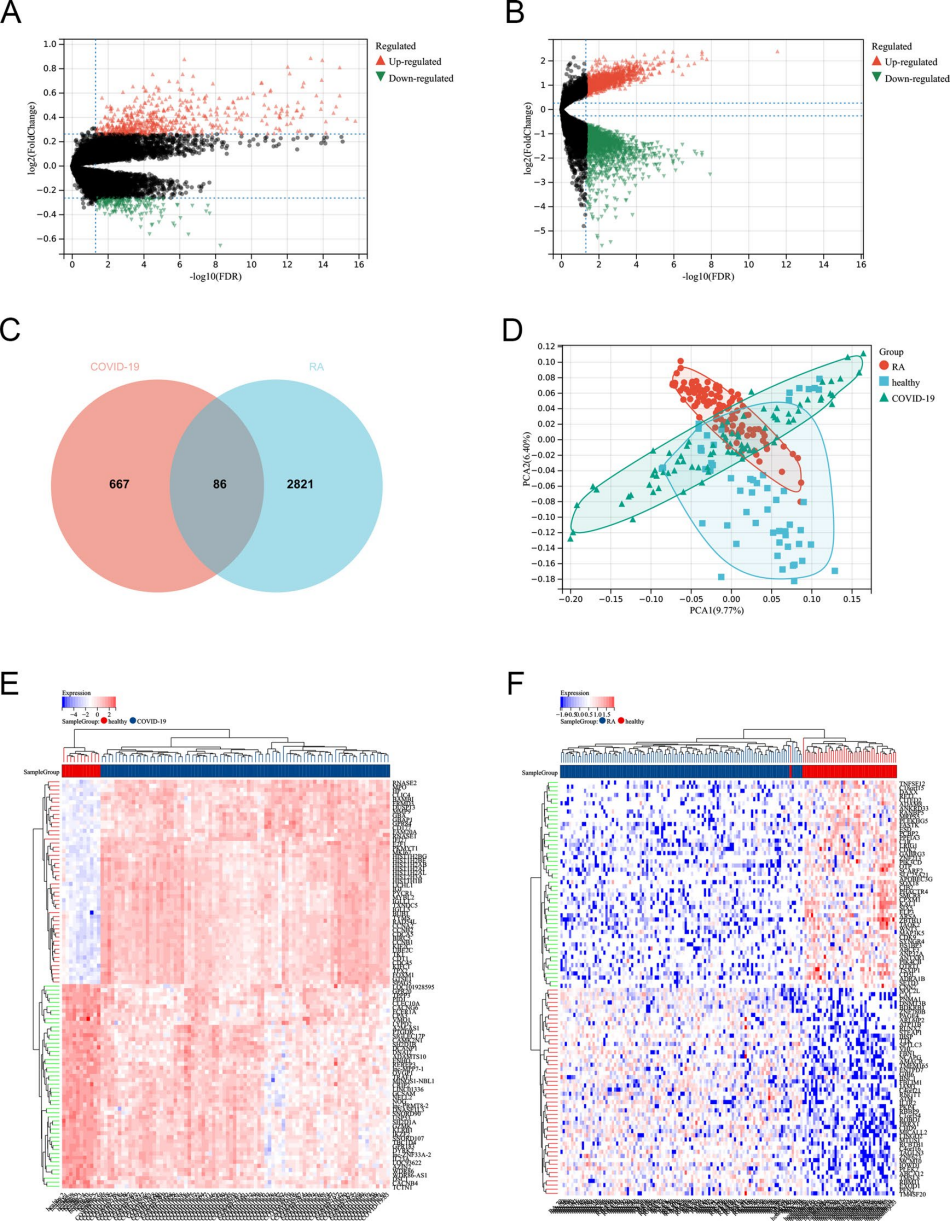
Differentially expressed genes identification. (**A**) Volcano plot of DEGs between COVID-19 and healthy (GSE213313); (**B**) Volcano plot of DEGs between RA and healthy (GSE17755); (**C**) Venn diagram shows the common DEGs between COVID-19 and RA; (**D**) PCA cluster plot of all samples; (**E**) Heatmap of the 100 most significant DEGs (GSE213313); (**F**) Heatmap of the 100 most significant DEGs (GSE17755)

After using the FDR < 0.05 and |log2 fold change (FC)| > 1.2 standard to screen for DEGs, 546 up-regulated genes and 207 down-regulated genes were found in the GSE213313 dataset, and 1316 up-regulated genes and 1591 down-regulated genes were found in the GSE17755 dataset (Fig. 6A-B). The Venn diagram shows that there are 86 common DEGs between the two (Fig. 6C). PCA clustering analysis of the dataset showed significant differences between RA, COVID-19, and normal controls, while there were similarities between RA and COVID-19 (Fig. 6D). The heatmap showed the 50 up-regulated genes and 50 down-regulated genes with the most significant differences (Fig. 6E-F).

### Enrichment analysis

**Fig. 7:**
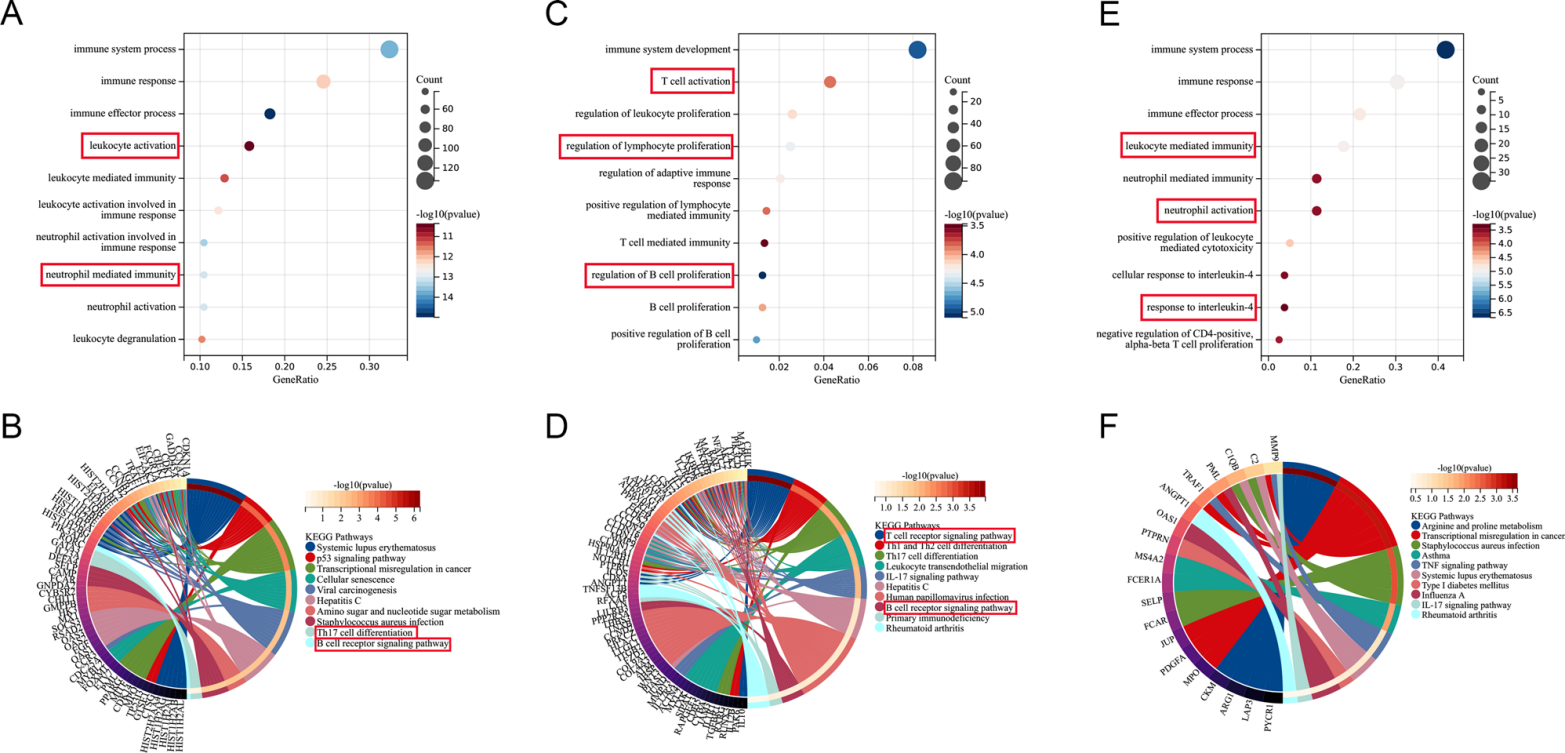
Enrichment analysis of DEGs. (**A**) GO function enrichment of DEGs between COVID-19 and healthy; (**B**) KEGG pathway enrichment of DEGs between COVID-19 and healthy; (**C**) GO function enrichment of DEGs between RA and healthy; (**D**) KEGG pathway enrichment of DEGs between RA and healthy; (**E**) GO functional enrichment of common DEGs; (**F**) KEGG pathway enrichment of common DEGs

The GO enrichment analysis of COVID-19 and control DEGs mainly focused on immune responses mediated by leukocytes and neutrophils. KEGG pathway analysis showed that systemic lupus erythematosus, viral carcinogenesis, and hepatitis C had statistical significance (Fig. 7A-B). The same analysis was performed on DEGs of RA and control. GO biological processes were significantly enriched in various lymphocyte proliferation, such as T cells, B cells. In addition, Th1, Th2, and Th17 cell differentiation, T cell and B cell receptor signaling were considered the most significant enrichment pathways (Fig. 7C-D). The common DEGs between the two was mainly related to various cell regulated immune and IL-4 responses. The most enrichment pathways include staphylococcus aureus infection and influenza A, systemic lupus erythematosus and type 1 diabetes mellitus (Fig. 7E-F).

#### Infiltration of immune cells

**Fig. 8:**
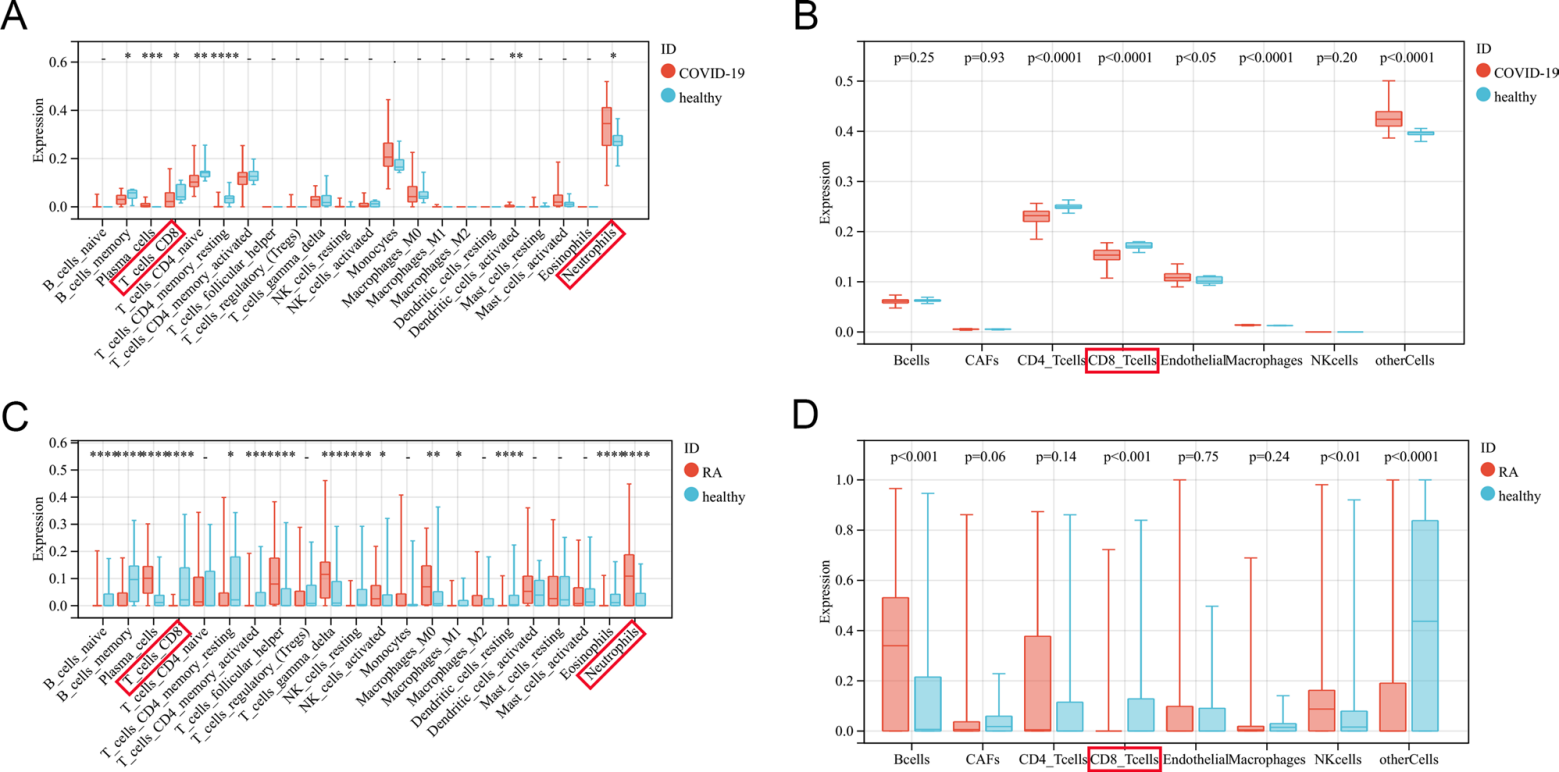
Evaluation of immune cell infiltration in COVID-19 and RA. (**A**) Violin diagram of COVID-19 and healthy immune cells using CIBERSORT algorithm; (**B**) Violin diagram of COVID-19 and healthy immune cells using EPIC algorithm; **(C**) Violin diagram of RA and healthy immune cells using CIBERSORT algorithm; (**D**) Violin diagram of RA and healthy immune cells using EPIC algorithm. (*p < 0.05, **p < 0.01, ***p < 0.001, ****p < 0.0001)

According to the CIBERSORT algorithm, compared to normal samples, COVID-19 samples contain a higher proportion of neutrophils and activated dendritic cells, while the proportion of B cells, CD4, and CD8 T cells is relatively low (Fig. 8A). The EPIC algorithm was validated, and under the standard of *p* < 0.05, the COVID-19 sample contained a high proportion of endothelial cells and macrophages, while the proportion of CD4 and CD8 T cells was relatively low (Fig. 8B). Similarly, the violin diagram showed that the RA group had more plasma cells, follicular helper T cells, activated NK cells, neutrophils and fewer CD8 T cells, M1 macrophages, eosinophils (Fig. 8C). Based on EPIC, the RA group has more B cells and NK cells, and relatively fewer CD8 T cells (Fig. 8D).

## Discussion

The respiratory tract is considered the primary site of SARS-CoV-2 infection, but many patients experience joint and muscle pain in specific areas, such as the waist, knee, ankle, etc. The severe cases did not disappear after recovery [20]. It is reported that new axial arthritis, peripheral arthritis and attachment point inflammation were detected in the imaging examination of patients with COVID-19. Positive SARS-CoV-2 IgG antibodies were also detected in the patient’s synovial fluid [21]. The etiology of inflammatory arthritis caused by this virus has not yet been elucidated, but there are many similarities with rheumatic diseases such as RA and SpA. Roongta reported RA cases with serum ACPA positivity after SARS-CoV-2 infection [22]. There were also reports of positive detection of rheumatoid factor and HLA-B27 in laboratory tests [23]. However, most studies are limited to case reports. COVID-19 has not yet been identified as the cause of rheumatoid arthritis [24]. In our meta-analysis, the prevalence of RA in COVID-19 patients did not significantly increase compared to the control population. Due to the limited number of studies included and the lack of large-scale prospective cohort studies or randomized controlled trials for evaluation, existing studies may draw biased conclusions. Therefore, we conducted a reverse meta-analysis and found that RA significantly increased the number of hospitalizations, severe infections, ICU hospitalizations, and deaths in COVID-19 patients. In addition, the antibody positive rate of RA patients after receiving COVID-19 vaccine was significantly lower than that of the control group, indicating that their immune response may be weaker. Topless found that RA patients are more likely to develop comorbidities such as asthma, chronic obstructive pulmonary disease, hypertension, and cardiovascular disease [25]. Therefore, it can be said that RA represents the potential threat of worsening results of COVID-19 and increases the risk of adverse outcomes of COVID-19.

In order to further study the relationship between COVID-19 and RA and minimize the impact of confounding factors on the association between COVID-19 and RA, we conducted MR analysis using GWAS summary statistics to obtain more reliable results. Genotypes are innate and are not affected by factors such as growth environment, economic status, or behavioral factors. The causal relationship based on MR research can provide favorable evidence for inferring the causal relationship between exposure factors and outcomes in situations where RCT cannot be implemented [26]. We did find a positive correlation between COVID-19 infection and RA. Therefore, there may be a causal relationship between COVID-19 infection and increased risk of RA, but COVID-19 does not directly cause RA, and the specific mechanism of this relationship still needs further research. After infection with COVID-19, the infected host will quickly execute innate and adaptive immune responses, which is the first line of defense against COVID-19 infection. Subsequently, CD4 + helper T cells promote the overall adaptive response by recruiting Th1 cells and supporting the differentiation of B lymphocytes to produce specific anti COVID-19 antibodies. CD8 + T cells also produce interferon to neutralize coronavirus [27]. RA has a highly similar abnormal immune response mechanism, and the role of CD4 + T cells in inducing chronic joint inflammation in RA patients has been revealed. It affects Th1 cells in synovial tissue to produce pro-inflammatory mediators, activate immune cells, and support B cell differentiation to produce autoantibodies [28]. In COVID-19 patients, DEGs are significantly enriched in immune responses mediated by white blood cells and neutrophils, as well as KEGG pathways such as systemic lupus erythematosus, indicating that the pathological mechanism of COVID-19 is closely related to extensive immune activation and inflammatory response. In RA patients, DEGs are mainly enriched in pathways such as T cell and B cell proliferation, as well as Th1, Th2, and Th17 cell differentiation, highlighting the abnormal activation of the adaptive immune system and chronic inflammatory response in RA. In our study, we also observed a higher proportion of neutrophils in COVID-19, while the proportion of B lymphocytes, CD4 and CD8 T lymphocytes was relatively low. RA share a higher proportion of neutrophils and a relatively lower proportion of CD8 T lymphocytes with COVID-19, but the difference is that B lymphocytes are relatively more abundant. Although according to MR analysis of immune cell phenotype, T cells and B cells are the main immune cells associated with COVID-19 and RA. However, enrichment analysis further revealed a greater correlation between RA and B cells, as well as T cells. COVID-19 is also associated with lymphocytes, but mainly with neutrophils and leukocytes. In COVID-19, the increase in neutrophils and decrease in lymphocyte count often indicate severe condition [29]. Previously, due to technological challenges, research on neutrophils was particularly difficult, but with the emergence of new methods, the role of neutrophils in RA is gradually being understood. In the joints of RA patients, especially in the early stages of the disease, neutrophils are abundant [30]. Neutrophils are also the main source of citrulline antigens, catalyzing the modification of arginine to citrulline [31].

In addition, we also observed the correlation between a variety of inflammatory mediators, such as interleukins, chemokines and tumor necrosis factors, and COVID-19 and RA. Interleukin is one of the inflammatory mediators most closely related to two diseases. The DEGs shared by RA and COVID-19 mainly involve cell regulated immune responses and IL-4 signaling pathways, further emphasizing the overlap between the two in immune inflammatory mechanisms. IL-13 is an inflammatory mediator obtained from MR analysis, while IL-4 and IL-17 is obtained through enrichment analysis. IL-13/IL-4 are potential therapeutic target shared by both diseases. Research has shown that increasing local IL-13 levels in joints can effectively suppress joint inflammation, thereby preventing and treating RA [32]. In COVID-19, IL-13 reduces the viral load and cell to cell transmission by reducing the expression of ACE2, effectively weakening the infection and replication ability of COVID-19 [33]. IL-4 exerts immunomodulatory effects by inhibiting the production of pro-inflammatory cytokine IL-17 in the synovium of RA patients, indicating its anti-inflammatory function in the chronic inflammatory stage [34]. However, in COVID-19, IL-4 may enhance immune response by promoting the secretion of inflammatory factors, helping the body resist the virus, indicating that it may have a pro-inflammatory effect during the acute infection stage [35]. These results indicate that there are many similarities in immune inflammatory characteristics between RA and COVID-19, but there are also differences.

## Conclusion

Our study established the correlation between COVID-19 and RA, and COVID-19 may lead to the progression of RA genetically. The immunotherapy methods of interleukins and immune cells in RA patients may also be a potential treatment for COVID-19.

## Electronic supplementary material

Below is the link to the electronic supplementary material.

Supplementary Material 1

Supplementary Material 2

Supplementary Material 3

Supplementary Material 4

Supplementary Material 5

Supplementary Material 6

Supplementary Material 7

Supplementary Material 8

## Data Availability

The data used to support the findings of this study are included within the article.
