# Supplementary material for: Immunoinflammatory evidence of rheumatoid arthritis caused by COVID-19
Source: Biol Res. 2025 Jun 10;58:38. doi: 10.1186/s40659-025-00620-7 (PMC12150480; doi:10.1186/s40659-025-00620-7)
Supplement: Supplementary file 1 — Supplementary Material 1 [file 40659_2025_620_MOESM1_ESM.docx]

1 Abdollahi, A. *et al.* The association between the level of serum 25(OH) vitamin D, obesity, and underlying diseases with the risk of developing COVID-19 infection: A case-control study of hospitalized patients in Tehran, Iran. *J Med Virol* **93**, 2359-2364, doi:10.1002/jmv.26726 (2021).

2 Ji, W. *et al.* Effect of Underlying Comorbidities on the Infection and Severity of COVID-19 in Korea: a Nationwide Case-Control Study. *J Korean Med Sci* **35**, e237, doi:10.3346/jkms.2020.35.e237 (2020).

3 Jung, Y., Kwon, M. & Choi, H. G. Association between previous rheumatoid arthritis and COVID-19 and its severity: a nationwide cohort study in South Korea. *BMJ Open* **11**, e054753, doi:10.1136/bmjopen-2021-054753 (2021).

4 Attauabi, M. *et al.* Coronavirus disease 2019, immune-mediated inflammatory diseases and immunosuppressive therapies - A Danish population-based cohort study. *J Autoimmun* **118**, 102613, doi:10.1016/j.jaut.2021.102613 (2021).

5 Boekel, L. *et al.* Antibody development and disease severity of COVID-19 in non-immunised patients with rheumatic immune-mediated inflammatory diseases: data from a prospective cohort study. *RMD Open* **8**, doi:10.1136/rmdopen-2021-002035 (2022).

6 Bournia, V. K. *et al.* Different COVID-19 outcomes among systemic rheumatic diseases: a nation-wide cohort study. *Rheumatology (Oxford)* **62**, 1047-1056, doi:10.1093/rheumatology/keac422 (2023).

7 Cooksey, R. *et al.* Shielding reduced incidence of COVID-19 in patients with inflammatory arthritis but vulnerability is associated with increased mortality. *Rheumatology (Oxford)* **61**, SI120-SI128, doi:10.1093/rheumatology/keac283 (2022).

8 Cordtz, R. *et al.* COVID-19 infection and hospitalization risk according to vaccination status and DMARD treatment in patients with rheumatoid arthritis. *Rheumatology (Oxford)* **62**, 77-88, doi:10.1093/rheumatology/keac241 (2022).

9 Eder, L. *et al.* Understanding COVID-19 Risk in Patients With Immune-Mediated Inflammatory Diseases: A Population-Based Analysis of SARS-CoV-2 Testing. *Arthritis Care Res (Hoboken)* **75**, 317-325, doi:10.1002/acr.24781 (2023).

10 England, B. R. *et al.* Risk of COVID-19 in Rheumatoid Arthritis: A National Veterans Affairs Matched Cohort Study in At-Risk Individuals. *Arthritis Rheumatol* **73**, 2179-2188, doi:10.1002/art.41800 (2021).

11 Kim, Y. *et al.* COVID-19 Outcomes in Myasthenia Gravis Patients: Analysis From Electronic Health Records in the United States. *Front Neurol* **13**, 802559, doi:10.3389/fneur.2022.802559 (2022).

12 Medeiros-Ribeiro, A. C. *et al.* Distinct impact of DMARD combination and monotherapy in immunogenicity of an inactivated SARS-CoV-2 vaccine in rheumatoid arthritis. *Ann Rheum Dis* **81**, 710-719, doi:10.1136/annrheumdis-2021-221735 (2022).

13 Topless, R. K. *et al.* Gout, Rheumatoid Arthritis, and the Risk of Death Related to Coronavirus Disease 2019: An Analysis of the UK Biobank. *ACR Open Rheumatol* **3**, 333-340, doi:10.1002/acr2.11252 (2021).

14 Wang, Y. *et al.* Increased Risk of COVID-19 in Patients With Rheumatoid Arthritis: A General Population-Based Cohort Study. *Arthritis Care Res (Hoboken)* **74**, 741-747, doi:10.1002/acr.24831 (2022).

15 Curtis, J. R. *et al.* Characteristics, Comorbidities, and Outcomes of SARS-CoV-2 Infection in Patients With Autoimmune Conditions Treated With Systemic Therapies: A Population-based Study. *J Rheumatol* **49**, 320-329, doi:10.3899/jrheum.210888 (2022).

16 D'Silva, K. M. *et al.* Clinical characteristics and outcomes of patients with coronavirus disease 2019 (COVID-19) and rheumatic disease: a comparative cohort study from a US 'hot spot'. *Ann Rheum Dis* **79**, 1156-1162, doi:10.1136/annrheumdis-2020-217888 (2020).

17 Figueroa-Parra, G. *et al.* Risk of severe COVID-19 outcomes associated with rheumatoid arthritis and phenotypic subgroups: a retrospective, comparative, multicentre cohort study. *Lancet Rheumatol* **4**, e765-e774, doi:10.1016/S2665-9913(22)00227-2 (2022).

18 Li, H. *et al.* Risk of COVID-19 Among Unvaccinated and Vaccinated Patients With Rheumatoid Arthritis: A General Population Study. *Arthritis Care Res (Hoboken)* **75**, 956-966, doi:10.1002/acr.25028 (2023).

19 Malek Mahdavi, A. *et al.* Factors associated with COVID-19 and its outcome in patients with rheumatoid arthritis. *Clin Rheumatol* **40**, 4527-4531, doi:10.1007/s10067-021-05830-4 (2021).

20 Pablos, J. L. *et al.* Clinical outcomes of hospitalised patients with COVID-19 and chronic inflammatory and autoimmune rheumatic diseases: a multicentric matched cohort study. *Ann Rheum Dis* **79**, 1544-1549, doi:10.1136/annrheumdis-2020-218296 (2020).

21 Raiker, R. *et al.* Outcomes of COVID-19 in patients with rheumatoid arthritis: A multicenter research network study in the United States. *Semin Arthritis Rheum* **51**, 1057-1066, doi:10.1016/j.semarthrit.2021.08.010 (2021).

22 Ermurat, S., Ayar, K., Avci, S. & Gursoy, V. Disease Course in Hospitalized COVID-19 Patients with and without Rheumatic Disease and Its Relationship with Immunosuppressive Drug Use. (2021).

23 Ge, E., Li, Y., Wu, S., Candido, E. & Wei, X. Association of pre-existing comorbidities with mortality and disease severity among 167,500 individuals with COVID-19 in Canada: A population-based cohort study. *PLoS One* **16**, e0258154, doi:10.1371/journal.pone.0258154 (2021).

24 Mena Vazquez, N. *et al.* Incidence and case fatality rate of COVID-19 in patients with inflammatory articular diseases. *Int J Clin Pract* **75**, e13707, doi:10.1111/ijcp.13707 (2021).

25 Zargaran, M., Movassaghi, S., Seyyedsalehi, M. S., Zendehdel, K. & Rostamian, A. Outcomes of coronavirus disease 19 patients with a history of rheumatoid arthritis: A retrospective registry-based study in Iran. *Int J Rheum Dis* **25**, 1196-1199, doi:10.1111/1756-185X.14405 (2022).

26 Alsaed, O. *et al.* Risk of Severe SARS-CoV-2 Infection in Patients with Autoimmune Rheumatic Diseases in Qatar: A Cohort Matched Study. *Qatar Med J* **2022**, 24, doi:10.5339/qmj.2022.24 (2022).

27 Farroni, C. *et al.* Kinetics of the B- and T-Cell Immune Responses After 6 Months From SARS-CoV-2 mRNA Vaccination in Patients With Rheumatoid Arthritis. *Front Immunol* **13**, 846753, doi:10.3389/fimmu.2022.846753 (2022).

28 Furer, V. *et al.* Predictors of Immunogenic Response to the BNT162b2 mRNA COVID-19 Vaccination in Patients with Autoimmune Inflammatory Rheumatic Diseases Treated with Rituximab. *Vaccines (Basel)* **10**, doi:10.3390/vaccines10060901 (2022).

29 Furukawa, H. *et al.* Anti-SARS-CoV-2 Spike Antibody Titers and Neutralizing Antibodies in Vaccinated Rheumatoid Arthritis Patients. *Vaccines (Basel)* **10**, doi:10.3390/vaccines10081365 (2022).

30 Mauro, D. *et al.* Serological Response to BNT162b2 Anti-SARS-CoV-2 Vaccination in Patients with Inflammatory Rheumatic Diseases: Results From the RHEUVAX Cohort. *Front Immunol* **13**, 901055, doi:10.3389/fimmu.2022.901055 (2022).

31 Picchianti-Diamanti, A. *et al.* ImmunosuppressiveTherapies Differently Modulate Humoral- and T-Cell-Specific Responses to COVID-19 mRNA Vaccine in Rheumatoid Arthritis Patients. *Front Immunol* **12**, 740249, doi:10.3389/fimmu.2021.740249 (2021).

32 Seyahi, E. *et al.* Antibody response to inactivated COVID-19 vaccine (CoronaVac) in immune-mediated diseases: a controlled study among hospital workers and elderly. *Rheumatol Int* **41**, 1429-1440, doi:10.1007/s00296-021-04910-7 (2021).

33 Vuilleumier, N. *et al.* Anti-SARS-CoV-2 mRNA vaccines as inducers of humoral response against apolipoprotein A-1? *Eur J Clin Invest* **52**, e13713, doi:10.1111/eci.13713 (2022).

34 Zhao, T. *et al.* Immunogenicity of Inactivated SARS-CoV-2 Vaccines in Patients With Rheumatoid Arthritis: A Case Series. *Front Public Health* **10**, 875558, doi:10.3389/fpubh.2022.875558 (2022).
