# Supplementary figures and images for: Immunoinflammatory evidence of rheumatoid arthritis caused by COVID-19
Source: Biol Res. 2025 Jun 10;58:38. doi: 10.1186/s40659-025-00620-7 (PMC12150480; doi:10.1186/s40659-025-00620-7)

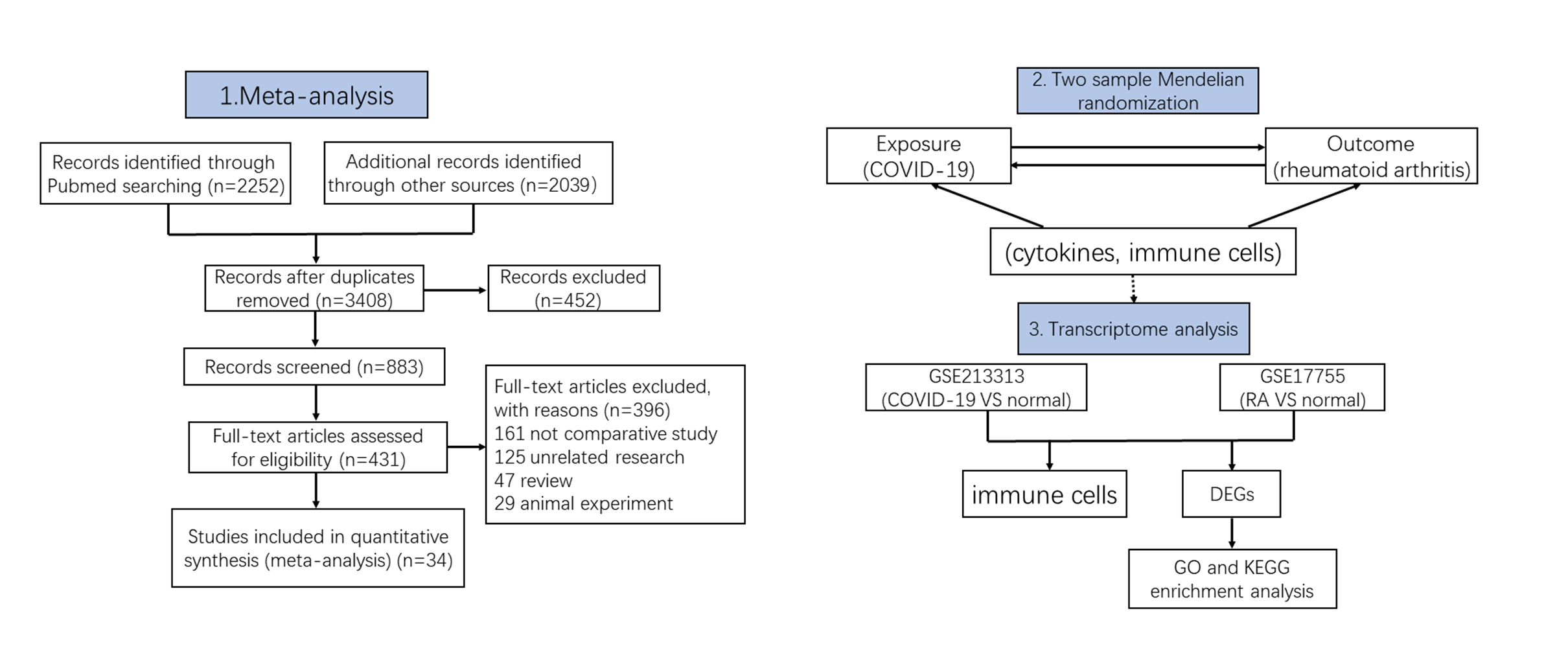

Supplement: Supplementary file 2 — Supplementary Material 2 [file 40659_2025_620_MOESM2_ESM.tif]

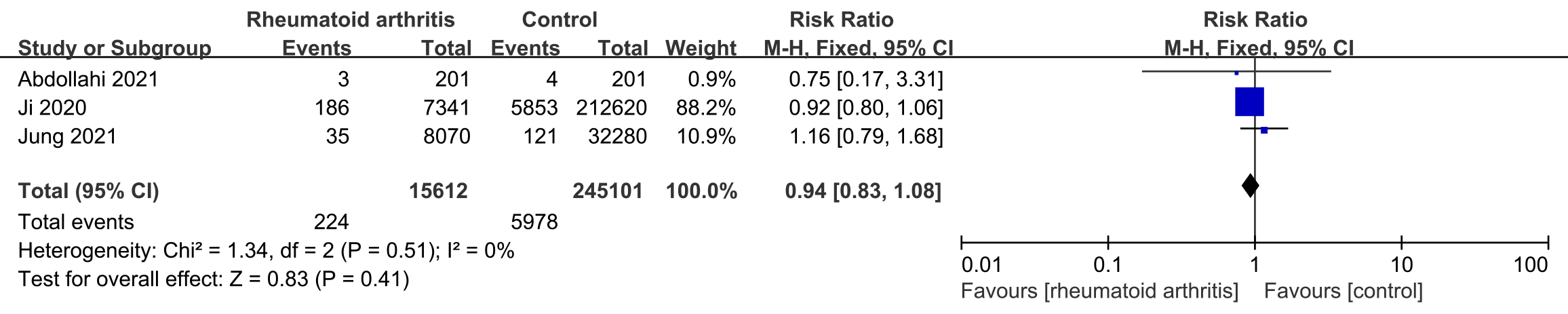

Supplement: Supplementary file 3 — Supplementary Material 3 [file 40659_2025_620_MOESM3_ESM.tif]

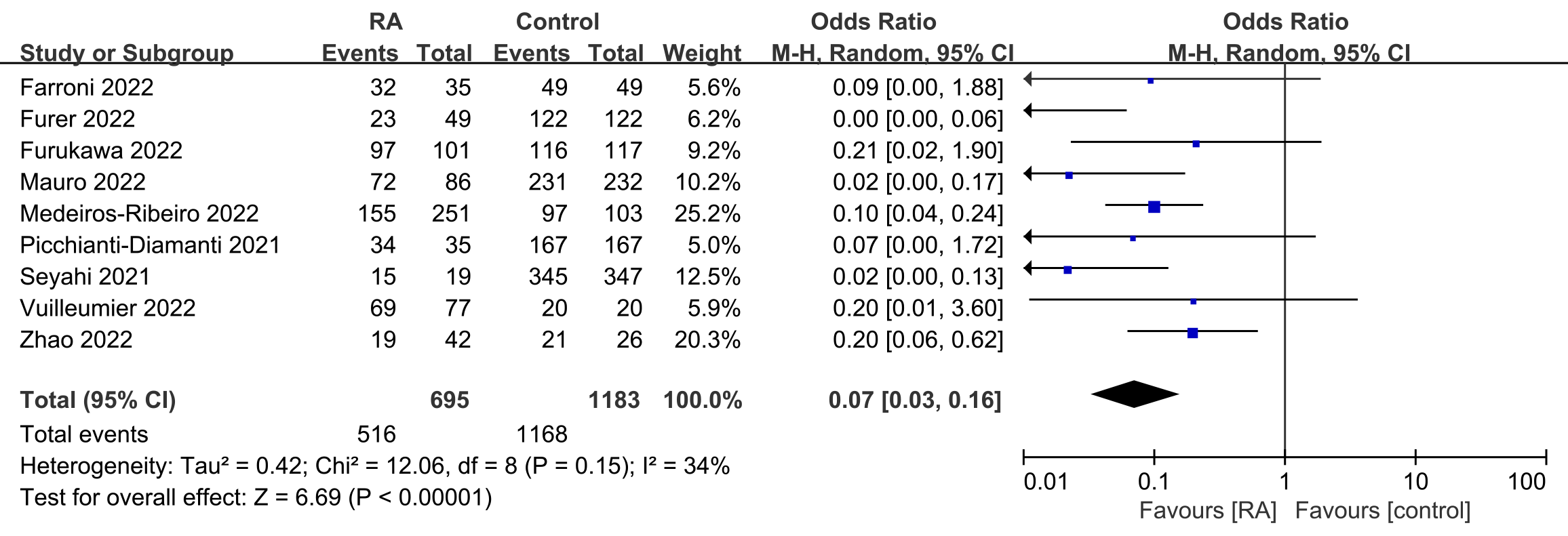

Supplement: Supplementary file 4 — Supplementary Material 4 [file 40659_2025_620_MOESM4_ESM.tif]

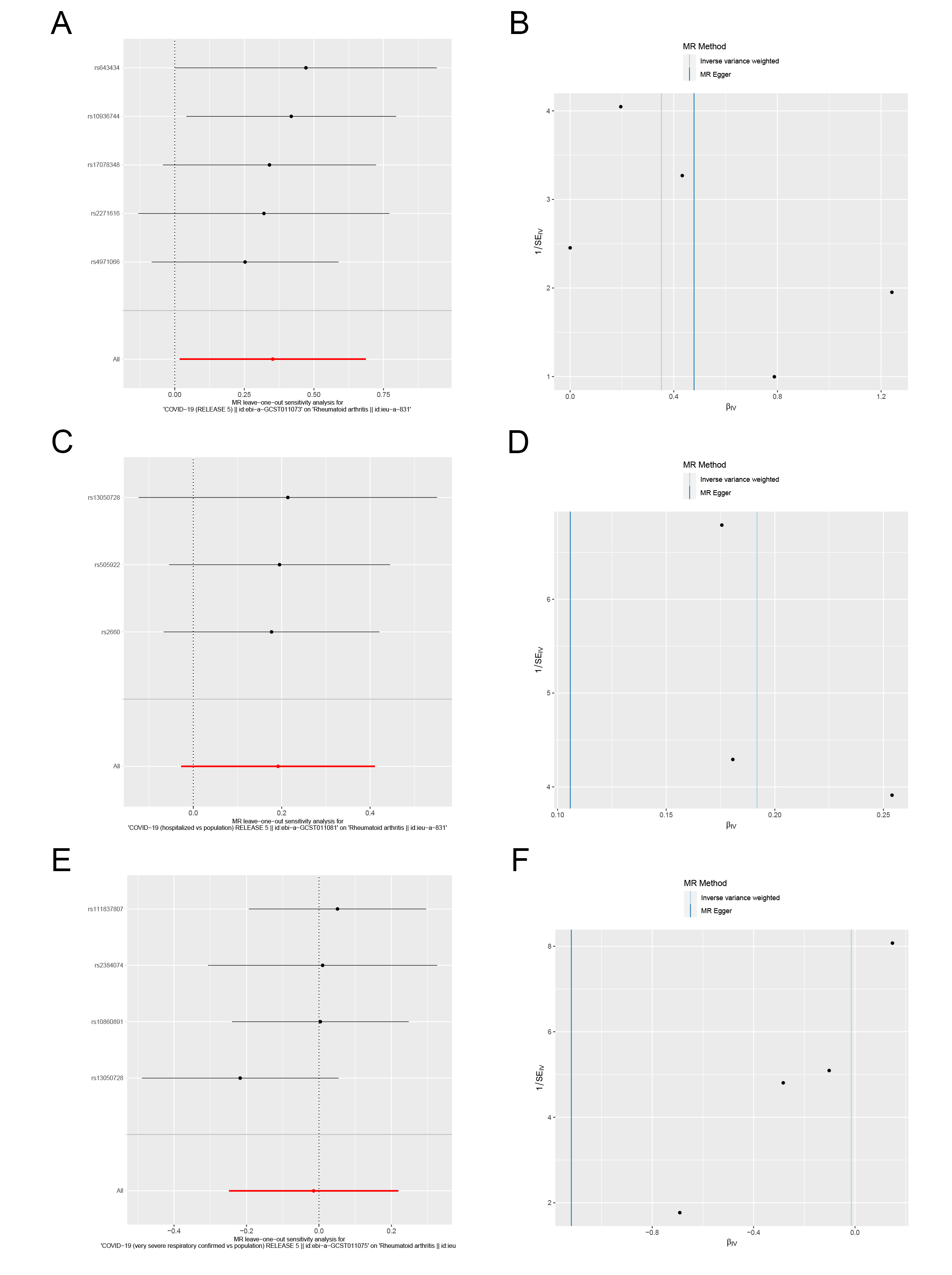

Supplement: Supplementary file 5 — Supplementary Material 5 [file 40659_2025_620_MOESM5_ESM.tif]

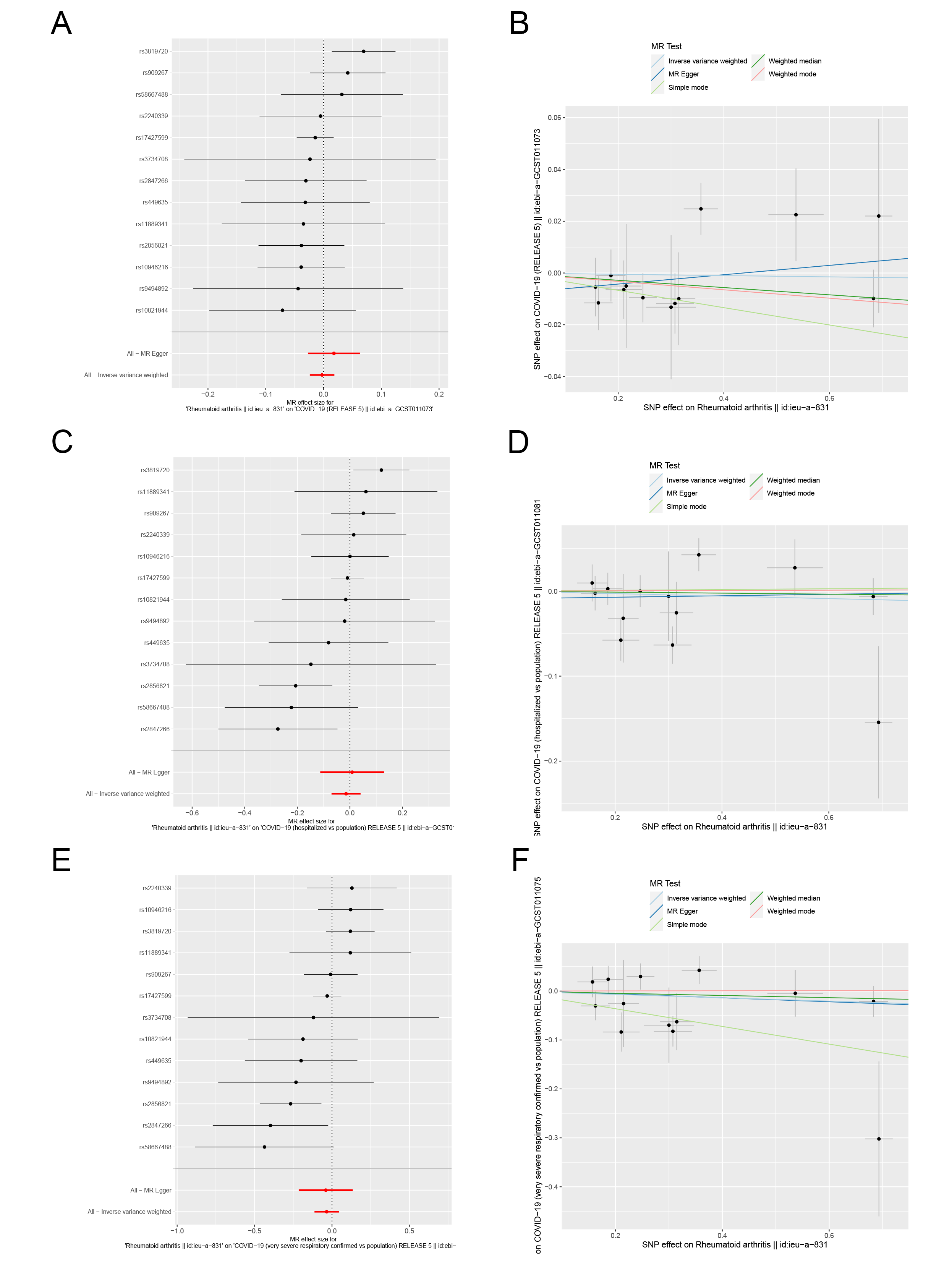

Supplement: Supplementary file 6 — Supplementary Material 6 [file 40659_2025_620_MOESM6_ESM.tif]

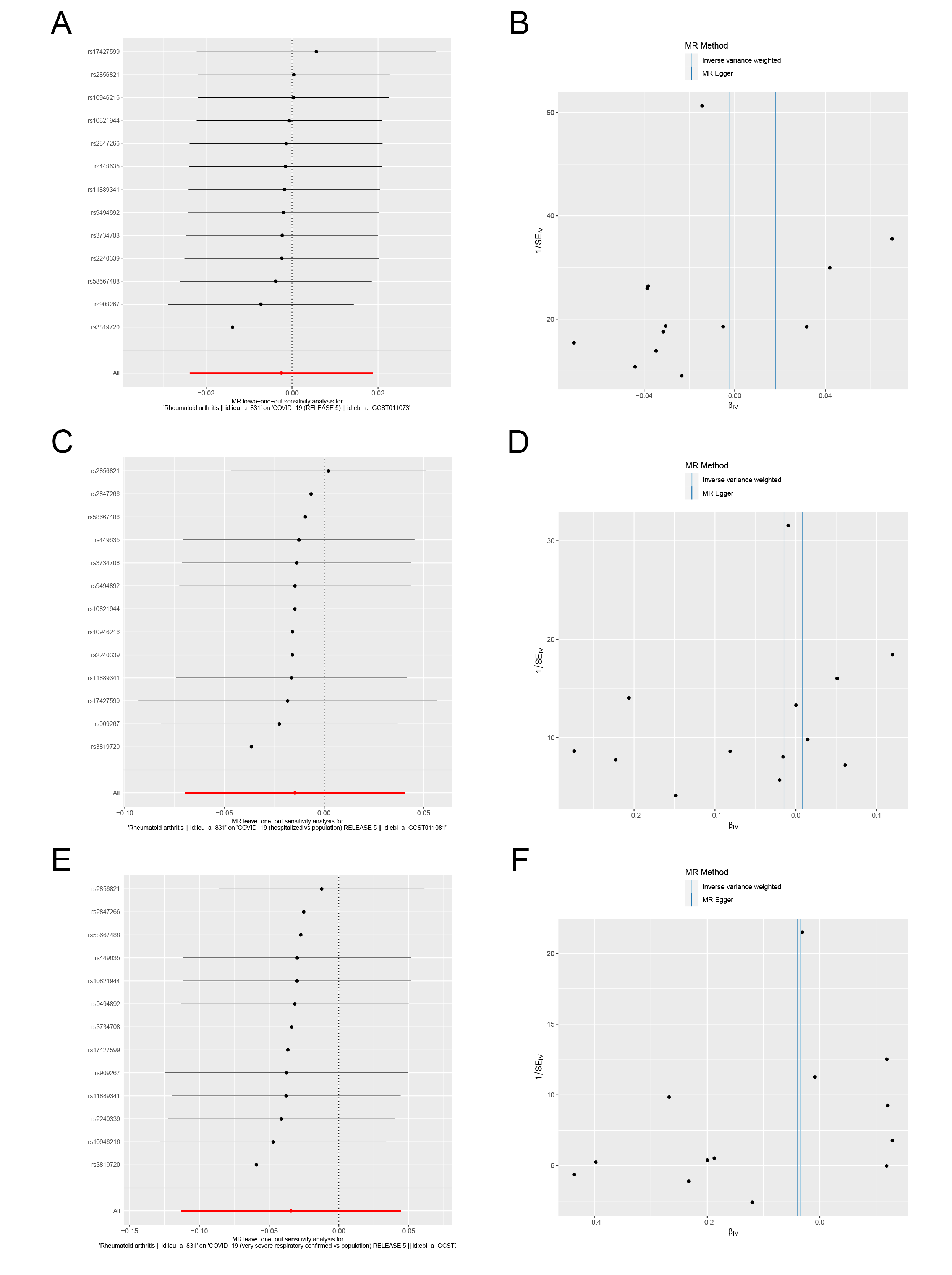

Supplement: Supplementary file 7 — Supplementary Material 7 [file 40659_2025_620_MOESM7_ESM.tif]
