# Supplementary material for: Immunoinflammatory evidence of rheumatoid arthritis caused by COVID-19
Source: Biol Res. 2025 Jun 10;58:38. doi: 10.1186/s40659-025-00620-7 (PMC12150480; doi:10.1186/s40659-025-00620-7)
Supplement: Supplementary file 8 — Supplementary Material 8 [file 40659_2025_620_MOESM8_ESM.docx]

**Supplemental Table 1** Detailed information on the summary GWAS results of COVID-19 and rheumatoid arthritis

| GWAS ID | Trait | Cases | Controls | Sample size | Year | Population |
| --- | --- | --- | --- | --- | --- | --- |
| ebi-a-GCST011073 | SARS-CoV-2 infection | 38984 | 1644784 | 1683768 | 2020 | European |
| ebi-a-GCST011081 | COVID-19 hospitalization | 9986 | 1877672 | 1887658 | 2020 | European |
| ebi-a-GCST011075 | Severe COVID-19 | 5101 | 1383241 | 1388342 | 2020 | European |
| ieu-a-831 | Rheumatoid arthritis | 4873 | 17642 | 22515 | 2014 | European |

**Supplemental Table 2** Trials included in the meta-analysis of rheumatoid arthritis in COVID-19 patients

| Author | Year | Country | COVID-19 | | | Control | | | Outcome  measure |
| --- | --- | --- | --- | --- | --- | --- | --- | --- | --- |
|  |  |  | number | age | gender | number | age | gender |  |
| Abdollahi | 2021 | Iran | 201 | 48±16.95 | NA | 201 | 46.34±13.5 | NA | 1 |
| Ji | 2020 | South Korea | 7341 | NA | 59.5 | 212620 | NA | 52.3 | 1 |
| Jung | 2021 | South Korea | 8070 | NA | 59.9 | 32280 | NA | 59.9 | 1 |

Age expressed as mean ± SD or median (IQR)

Gender expressed as percentage of women

Outcome measure: 1 represents the number of patients with rheumatoid arthritis of COVID-19 and the control. 2 represents the number of SARS-CoV-2 infection in patients with rheumatoid arthritis and control. 3 represents the number of COVID-19 hospitalizations in patients with rheumatoid arthritis and control. 4 represents the number of COVID-19 deaths in patients with rheumatoid arthritis and control. 5 represents the number of COVID-19 severe infections in patients with rheumatoid arthritis and control. 6 represents the number of COVID-19 ICU hospitalizations in patients with rheumatoid arthritis and control. 7 represents the number of antibody positive patients with rheumatoid arthritis and control after COVID-19 vaccine.

**Supplemental Table 3** Trials included in the meta-analysis of COVID-19 in patients with rheumatoid arthritis

| Author | Year | Country | Rheumatoid arthritis | | | Control | | | Outcome  measure |
| --- | --- | --- | --- | --- | --- | --- | --- | --- | --- |
|  |  |  | number | age | gender | number | age | gender |  |
| Attauabi | 2021 | Denmark | 5051 | NA | NA | 583788 | NA | NA | 2 |
| Boekel | 2022 | Netherlands | 2678 | NA | NA | 1102 | 55±14 | 68.9 | 2,3 |
| Bournia | 2022 | Greece | 62985 | NA | NA | 314925 | NA | NA | 2,3,4 |
| Cooksey | 2022 | Britain | 56914 | NA | NA | 2760442 | NA | NA | 2,3 |
| Cordtz | 2022 | Denmark | 27903 | 67.8(57-76) | 71.3 | 557415 | 67.9(58-76) | 71.3 | 2,4 |
| Eder | 2021 | Canada | 179701 | NA | NA | 898427 | NA | NA | 2 |
| England | 2021 | America | 33886 | 67.8±11.1 | 15.5 | 33886 | 67.8±11.1 | 15.5 | 2,6 |
| Ji | 2020 | South Korea | 6039 | NA | NA | 213922 | NA | NA | 2,6 |
| Kim | 2022 | South Korea | 511 | NA | 83.4 | 128609 | NA | NA | 2 |
| Medeiros-Ribeiro | 2022 | Brazil | 260 | 59(50-65) | 90.4 | 104 | 58(50-64) | 90.4 | 2,7 |
| Topless | 2021 | Britain | 5409 | NA | 71.6 | 467730 | NA | 55.1 | 2,4 |
| Wang | 2022 | Britain | 17268 | 64.9±13.5 | 71.2 | 1616600 | 53.3±16.8 | 54.0 | 2 |
| Curtis | 2022 | America | 2727 | NA | NA | 311563 | 49(18-89) | 57.1 | 3,4,5 |
| D’Silva | 2020 | America | 19 | NA | NA | 104 | 63.1±14.9 | 69.2 | 3 |
| Figueroa-Parra | 2022 | America | 582 | 62±14 | 72.3 | 2875 | 61±14 | 72.5 | 3,4,6 |
| Li | 2022 | Britain | 15901 | 64.8±13.7 | 70.8 | 1558423 | 47.9±17.2 | 48.9 | 3,4 |
| Mahdavi | 2021 | Iran | 128 | 52.3±13.9 | 83.6 | 92 | 48.4±16.2 | 42.4 | 3,4,5 |
| Pablos | 2020 | Spain | 65 | NA | NA | 228 | 65(53-77) | 58.3 | 3,4,5,6 |
| Raiker | 2021 | America | 9730 | 61.1±15.3 | 74.8 | 656979 | 47.6±18.7 | 55.0 | 3,4,5,6 |
| Ermurat | 2021 | Turkey | 23 | NA | NA | 1848 | 54(18-99) | 50.9 | 4 |
| Ge | 2021 | Canada | 1536 | NA | NA | 165964 | NA | NA | 4 |
| Jung | 2021 | South Korea | 35 | NA | 77.1 | 8035 | NA | 59.8 | 4,6 |
| Mena Vazquez | 2021 | Spain | 14 | NA | NA | 515 | NA | NA | 4 |
| Zargaran | 2022 | Iran | 38 | 59.9±11.9 | 76.3 | 2216 | 57.7±16.9 | 40.8 | 4,5 |
| Alsaed | 2022 | Qatar | 57 | NA | NA | 389 | 43.38±12.16 | 56.3 | 6 |
| Farroni | 2022 | Italy | 35 | 59(55-66) | 80.0 | 49 | 51(45-56) | 79.6 | 7 |
| Furer | 2022 | Israel | 49 | NA | NA | 122 | 50.83±14.64 | 64.8 | 7 |
| Furukawa | 2022 | Japan | 101 | 71.3±10.7 | 77.2 | 117 | 39.5±12.6 | 66.7 | 7 |
| Mauro | 2022 | Italy | 86 | NA | NA | 232 | 57 | 69.4 | 7 |
| Picchianti-Diamanti | 2021 | Italy | 35 | 59(55-65) | 77.1 | 167 | 42(32-53) | 71.3 | 7 |
| Seyahi | 2021 | Turkey | 19 | NA | NA | 347 | NA | NA | 7 |
| Vuilleumier | 2022 | Switzerland | 77 | 63.6±12.7 | 59.7 | 20 | 44.8±13.9 | 75.0 | 7 |
| Zhao | 2022 | China | 42 | 56(45.5-60) | 71.4 | 26 | 44.5(36-53) | 42.3 | 7 |

**Supplemental Figure legends:**

**Supplemental Figure 1.** Flow diagram showing the process of meta-analysis inclusion and exclusion.

**Supplemental Figure 2.** Forest plot of RA prevalence in COVID-19 patients.

**Supplemental Figure 3.** Forest plot of antibody positive patients with RA after COVID-19 vaccine.

**Supplemental Figure 4.** Results of COVID-19 and RA leave-one-out method sensitivity analysis and funnel plots. (A) Leave-one-out sensitivity analysis for the effect of SARS-CoV-2 infection on RA; (B) Funnel plot for the effect of SARS-CoV-2 infection on RA; (C) Leave-one-out sensitivity analysis for the effect of COVID-19 hospitalization on RA; (D) Funnel plot for the effect of COVID-19 hospitalization on RA; (E) Leave-one-out sensitivity analysis for the effect of severe COVID-19 on RA; (F) Funnel plot for the effect of severe COVID-19 on RA.

**Supplemental Figure 5.** Forest plot and scatter plot for the causal relationship between RA and COVID-19 using different MR methods. (A) Forest plot of the causal relationships between RA and SARS-CoV-2 infection; (B) Scatter plot of the causal relationships between RA and SARS-CoV-2 infection; (C) Forest plot of the causal relationships between RA and COVID-19 hospitalization; (D) Scatter plot of the causal relationships between RA and COVID-19 hospitalization; (E) Forest plot of the causal relationships between RA and severe COVID-19; (F) Scatter plot of the causal relationships between RA and severe COVID-19. The slope of each line corresponds to the causal estimates for each method.

**Supplemental Figure 6.** Results of RA and COVID-19 leave-one-out method sensitivity analysis and funnel plots. (A) Leave-one-out sensitivity analysis for the effect of RA on SARS-CoV-2 infection; (B) Funnel plot for the effect of RA on SARS-CoV-2 infection; (C) Leave-one-out sensitivity analysis for the effect of RA on COVID-19 hospitalization; (D) Funnel plot for the effect of RA on COVID-19 hospitalization; (E) Leave-one-out sensitivity analysis for the effect of RA on severe COVID-19; (F) Funnel plot for the effect of RA on severe COVID-19.
